# Catecholaminergic activity among young adults after high phosphorus intake during childhood and adolescence

**DOI:** 10.1007/s42000-025-00735-2

**Published:** 2025-11-12

**Authors:** Luciana Peixoto Franco, Seyedeh-Masomeh Derakhshandeh-Rishehri, Ute Nöthlings, Hermann Kalhoff, Mirko Peitzsch, Stefan A. Wudy, Thomas Remer

**Affiliations:** 1 DONALD Study Center, Nutritional Epidemiology, Institute of Nutrition and Food Science, University of Bonn, Dortmund, 44225 Heinstück 11, Germany; 2 Institute of Nutrition and Food Sciences, Nutritional Epidemiology, University of Bonn, Bonn, Germany; 3 Research Department of Child Nutrition, St. Josef-Hospital, University Hospital of Pediatrics and Adolescent Medicine, Ruhr-University Bochum, Bochum, Germany; 4 Pediatric Clinic Dortmund, Dortmund, Germany; 5 Institute of Clinical Chemistry and Laboratory Medicine, Faculty of Medicine Carl Gustav Carus at the Technische, University Hospital, Universität Dresden, Dresden, Germany; 6 Laboratory for Translational Hormone Analytics, Peptide Hormone & Immunoassay Unit, Pediatric Endocrinology & Diabetology, Center of Child and Adolescent Medicine, Justus Liebig University, Giessen, Germany

**Keywords:** 24-h urinary biomarkers, Catecholamines, Children, Epinephrine, Norepinephrine, Phosphorus intake, Phosphate excretion

## Abstract

**Purpose:**

Elevated catecholamine secretion has been reported after high dietary phosphate intake in experimental studies in animals and humans. This study thus aimed to examine the prospective relationship between long-term dietary phosphorus intake during childhood and adolescence—assessed via 24-h urinary phosphate excretion—and catecholamine levels in adulthood.

**Methods:**

A total of 159 healthy participants of the DONALD Study (Dortmund, Germany) were examined, who had repeatedly provided 24-h urine samples between ages 3 and 17 years and from whom a 24-h specimen was collected again in young adulthood (ages 18–35). In the adult samples, urinary free epinephrine (EPI), norepinephrine (NE) and the O-methylated EPI- and NE-metabolites metanephrine and normetanephrine were quantified using LC-MS/MS. Phosphate was measured ion chromatographically. Individual means of standard deviation scores were calculated for urinary phosphate and further biomarker excretions as well as for anthropometric data longitudinally determined between 3 and 17 years. Multivariable linear regression was used to investigate associations between pre-adulthood phosphate and adult catecholamine excretions.

**Results:**

After fully adjusting for growth- and adulthood-related covariates, only females’ renal excretions of EPI (p=0.030) and NE (p=0.040) were associated significantly with pre-adulthood phosphate excretion. In line with a disease-free, relatively continuous adrenal-medullary production of O-methylated metabolites, no association at all was seen for metanephrine and normetanephrine.

**Conclusion:**

Our study provides biomarker-based evidence that habitual high dietary phosphorus intake during childhood and adolescence may be related to elevated catecholaminergic activity in adulthood, at least in females, potentially contributing in the long term to endocrine-metabolic-related neuronal and cardiovascular disorders.

## Introduction

The catecholamines epinephrine (EPI) and norepinephrine (NE) are essential neurotransmitters and hormones that are released both from sympathetic neuronal nerve terminals throughout the body and from chromaffin medullary cells in the adrenal gland. Sympathetic neurons release NE locally in the tissues they innervate, whereas chromaffin cells in the adrenal medulla release EPI and NE into the circulation, thus augmenting the metabolic rate of virtually every cell in the organism [1]. EPI secreted by the adrenal medulla constitutes approximately 75% of the total amount of the gland’s catecholamine production [1]. Both catecholamines regulate a wide range of physiological processes that are closely related to the physiological maintenance of the cardiovascular, respiratory, and metabolic functions of the body. Accordingly, chronic dysregulation of EPI and NE causes a variety of metabolic, endocrine, cardiovascular, neurological, and psychiatric disorders [2, 3].

**Fig. 1:**
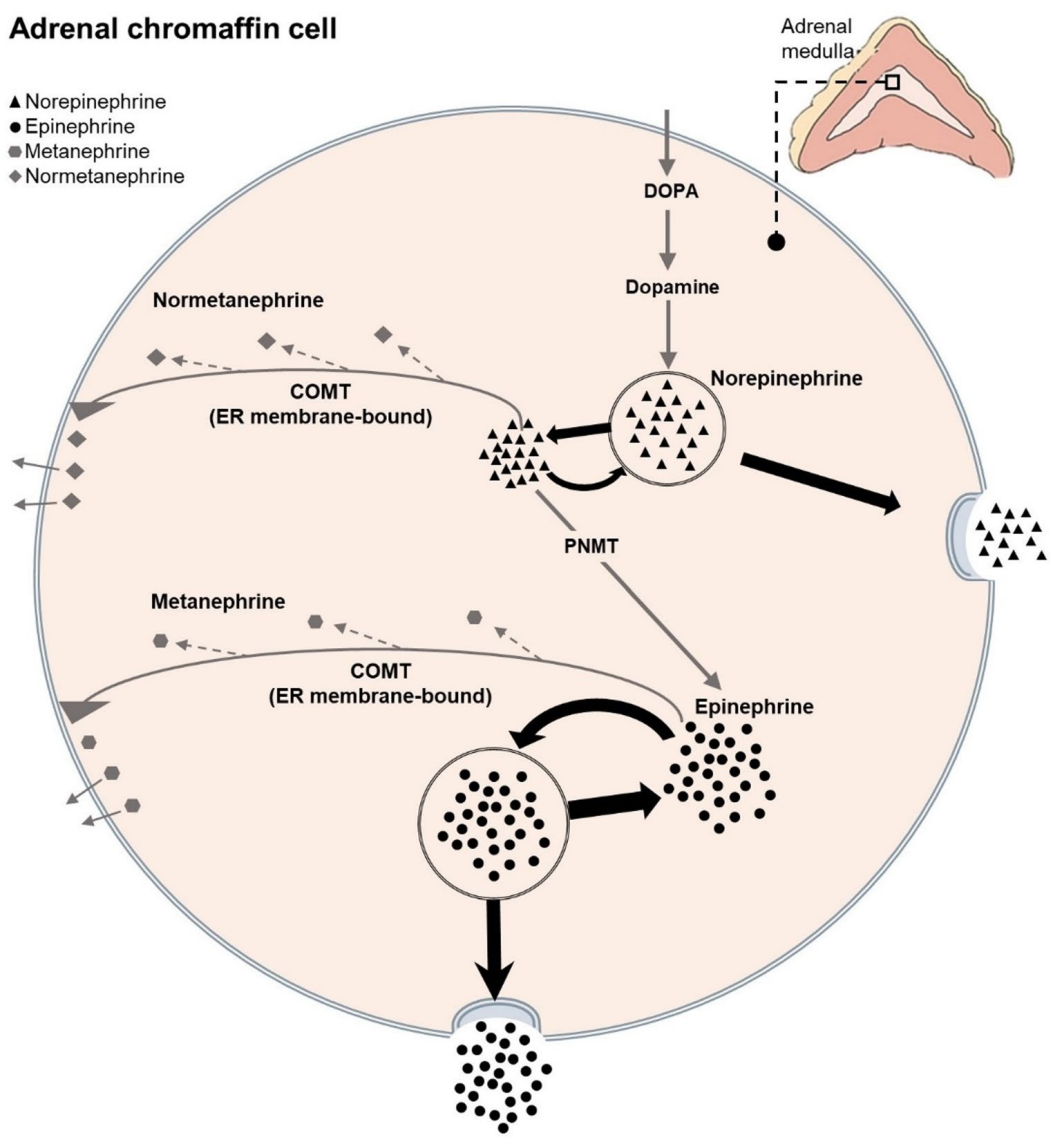
Schematic representation of catecholamine synthesis and metabolism in a chromaffin cell of the adrenal medulla. Dopamine taken up from medullary cells’ cytoplasma into cells’ storage vesicles is converted to norepinephrine (NE), which, after release from the vesicles, is methylated by the cytoplasmatic enzyme phenylethanolamine N-methyltransferase (PNMT) to produce epinephrine (EPI) [5]. Both NE and EPI are stored in vesicles and released into the bloodstream in response to respective physiological or respective stress stimuli. Catechol-O-metyltransferase (COMT), an endoplasmic reticulum (ER) membrane-bound enzyme, catalyzes the metabolism of NE and EPI to normetanephrine and metanephrine, respectively. Notably, metabolite production via COMT remains relatively stable and produces relatively small increases in plasma metanephrines, even after clear elevations of sympathoadrenal activity, i.e., stimulated EPI and NE secretion [3]

Within the adrenal medulla, catecholamines are predominantly stored in specific organelles known as chromaffin granules. Apart from containing high concentrations of EPI and NE, these granules also store considerable amounts of nucleotides, including diadenosine polyphosphates, adenosine triphosphate (ATP), ascorbic acid, calcium, neuropeptides, and proteins [4]. Within the chromaffin granules, the stored catecholamines are in part physically bound to the high-energy activated phosphate donor ATP [4]. Filled with all the latter actively accumulated solutes, these granules constitute the secretory vesicles which, after receiving a stimulus, are translocated to the plasmalemma where fusion pore formation and efficient secretion of the catecholamines take place [1, 4] (Fig. 1).

Under severe stress conditions, e.g., metabolic stress, intensive and excessive exercise, intense fear, or a prolonged state of struggle, adrenal medullary cells can, in a near-explosive manner, release EPI and NE into the circulation, thus establishing and regulating the “fight-or-flight” response [1, 4]. Consequences, among others, include pupil and bronchiole dilation as well as increased myocardial contraction, heart rate, blood pressure, and blood glucose levels, all thereby preparing the entire organism, including the skeletal muscles, to fight or else flee danger [1].

However, moderate increases in blood pressure and glycemia along with reductions in glucose tolerance do not occur only due to severe acute stress but are also observed following small elevations of EPI in the physiological or lower pathophysiological range [6–8]. Among potential nutritional influences on the sympathetic adrenomedullary system, high dietary intakes of phosphate have recently been reported to raise catecholamine levels in healthy human subjects [9] as well as in animal experiments in mice [10]. In the human study, over a total period of 11 weeks, healthy young adults ingested controlled diets providing either a high phosphate content within the range observed in US-American adults or a low phosphate intake [9]. The animal experiment was also a long-term study during which a standard mice diet with or without a surplus of phosphate was fed for 14 months [10].

Whether a high dietary phosphorus intake ingested habitually over many years may affect sympathoadrenergic activity in healthy humans in the long run is not known. We therefore aimed to examine, based on biomarkers, the prospective relationship between habitual phosphorus intake during childhood and adolescence and 24-h excretion rates of catecholamines in later adulthood.

## Materials and methods

### Study population

In this prospective longitudinal study, healthy children and adolescents aged 3 to 17 years who participated until adulthood in the ongoing Dortmund Nutritional and Anthropometric Longitudinally Designed (DONALD) study were examined. The DONALD study started as an open cohort in 1985 in Dortmund, Germany, and has since then prospectively collected data on anthropometrics, diet, metabolism, and growth and development from infancy to adulthood in healthy volunteers [11].

After the age of 2 years, yearly examinations of children are performed, including medical anamnesis, dietary recordings, anthropometric measurements, and 24-h urine collections (the latter at or after age 3). Participants older than 18 years are invited to provide a fasting blood sample (typically every 5 years). All evaluations are performed with parental and older children’s written consent. The study protocol was approved by the ethics committee of the University of Bonn, Germany (approval numbers. 098/06 and 185/20) and conducted according to the guidelines of the Declaration of Helsinki.

Participants with a minimum of five eligible 24-h urine collections, i.e., at least two collected within ages 3–8 years and at least three collected during adolescence (ages 9–17 years) were initially considered, resulting in 578 participants. Those participants who had at least five 24-hour urine samples with available measured values of PO4 excretion between ages 3 and 17 years and from whom an adult blood sample was provided were further selected (*n* = 343). Finally, a total of 159 participants were included in the study, all of whom – between ages 18 and 35 – had collected a 24-h urine sample around the time of the blood sample donation. None of these 159 subjects were using antihypertensives, antihypotensives, beta-blockers, calcium channel blockers, RAAS inhibitors, corticosteroids, or diuretics. Outcomes of interest, i.e., catecholamines, were measured in these adulthood 24-h urine samples.

### Anthropometric measurements

Anthropometric measurements were performed by trained nurses following standardized procedures, with participants dressed in underwear and barefoot [12, 13]. Standing height was measured to the nearest 0.1 cm with a digital wall-mounted stadiometer (Harpenden, Holtain Ltd, Crymych, UK), while body weight was determined to the nearest 0.1 kg using an electronic scale (Seca 753E; Seca Weighing and Measuring Systems, Hamburg, Germany). Height and body weight measurements were then used to calculate body mass index (BMI) and body surface area (BSA), according to the formulas: BMI (kg/m²) = weight/height^2^ and BSA (m²) = 0.007184 x height (cm)^0.725^ x weight (kg)^0.425^, as described by Du Bois and Du Bois [14].

Skinfold thickness measurements were conducted bilaterally at the triceps and subscapular, with precision to the nearest 0.1 mm, using a Holtain caliper. Body fat percentage (BF%) was subsequently estimated using the skinfold data and the equations of Slaughter et al. [15]. Fat mass index (FMI) was calculated by dividing fat mass by height squared (kg/m^2^).

### Urinary measurements

Each child and their parents were provided with personal and written instructions on how to collect the 24-h urine sample according to standardized procedures. Urine samples were self-collected at home using Extran-cleaned (Extran, MA03; Merck), preservative-free, 1-L plastic containers. The collected samples were stored at −18 to −20° C until thawed for analysis [16].

Urinary creatinine concentrations were quantified with a creatinine analyzer (Beckman-2; Beckman Instruments, Fullerton, CA, USA), based on the kinetic Jaffé procedure. Samples with daily creatinine excret**i**on levels below 0.1 mmol/kg were excluded from the analysis [17] to reduce potential errors in urine collection. Urinary urea levels were determined using the urease-Berthelot method (Randox Laboratories, Crumlin, UK). Uric acid concentrations (mmol/L) were assessed employing the uricase method using the Uric Acid plus kit (Roche Diagnostics GmbH, Mannheim, Germany) [16].

The alkalizing components of the urinary potential renal acid load (uPRAL), i.e., the mineral cations sodium, potassium, calcium, and magnesium were quantified using flame atomic absorption spectrometry (Perkin Elmer 1100 Spectrometer; Perkin Elmer, Überlingen, Germany). Urinary excretion rates of the anions phosphate, sulfate, and chloride were determined using a Dionex 2000i/SP ion chromatograph containing an ion Pac AS4A column (Dionex GmbH, Idstein, Germany). The uPRAL was calculated according to the formula:

- uPRAL = Chloride (mmol/d) + sulfate (mmol/d) x 2 + phosphate (mmol/d) x 1.8 – sodium (mmol/d) – potassium (mmol/d) – magnesium (mmol/d) x 2 – calcium (mmol/d) x 2) [18]. The ion excretion in mmol/d were converted to milliequivalents per day (mEq/d) by multiplying with the respective ionic valence [19].

Urinary free catecholamines, i.e., epinephrine, norepinephrine, and their metabolites metanephrine and normetanephrine, were quantified using liquid chromatography-tandem mass spectrometry (LC-MS/MS). Measurements were performed at the Institute of Clinical Chemistry and Laboratory Medicine, Dresden, Germany, based on a method described earlier [20], with minimal changes regarding the mass spectrometric detection system now using a QTRAP 6500+ (Sciex). 

### Blood measurements

Venous blood samples (< 20 ml) were collected after an overnight fast, centrifuged promptly at 4 °C within 15 min, and stored at −80 °C.

Plasma insulin concentrations were quantified with an immunoradiometric assay (IRMA; DRG Diagnostics, Marburg, Germany) and insulin resistance was evaluated using the homeostasis model assessment (HOMA-IR) [21].

Serum levels of uric acid, urea, glucose, LDL, and HDL cholesterol, as well as plasma concentrations of albumin, creatinine, triglycerides and phosphate were analyzed using a Roche/Hitachi Cobas c311 analyzer (Roche diagnostics, Mannheim, Germany) at the clinic laboratory of the Pediatric Clinic in Dortmund, Germany.

### Statistical analysis

All statistical analyses were conducted using SAS statistical software (SAS Institute Inc., Cary, NC, USA; version 9.2), with significance set at p-value < 0.05. The distribution of all variables was examined using the Shapiro-Wilk test and Q-Q plot.

Descriptive data of the participants are presented as means (± SD) for normally distributed variables and as medians (25th, 75th percentiles) for non-normally distributed variables. Differences in phosphate excretion between youth and adulthood assessments were analyzed using a paired t-test for normally distributed data. Furthermore, differences in catecholamine excretion between males and females were tested using an independent t-test, assuming normal distribution of the data. HOMA-IR values were log_10_ transformed in all regression models to normalize the distribution.

Anthropometric measurements and all pre-adulthood 24-h urinary biomarkers were internally standardized (mean = 0, SD = 1) by sex and age and the standardized deviation scores (SDS) were averaged as means of all individually available measurement points for each participant. The anthropometric and 24-h urinary excretion data from the growth period were then included in the analyses as the respective individual arithmetic means of SDS.

To examine the prospective associations of long-term high phosphorus intake (assessed by PO4 excretion) in childhood and adolescence with the adulthood outcomes—specifically epinephrine, norepinephrine, and their metabolites metanephrine and normetanephrine— multivariable linear regression models were conducted (PROC GLM in SAS). The assumptions for multiple linear regression models, including normal distribution of residuals, linearity, absence of multicollinearity, and homoscedasticity, were tested and not violated. Although diet-by-sex interactions were not observed for any of the catecholamines examined, sex-stratified regressions were conducted to account for the fact that males’ and females’ catecholamine responses have been reported to differ depending on the stressor [22–24].

To be included in the final sex-stratified statistical model, each potential covariate was tested separately using stepwise regression and further backward elimination. Covariates were included in the regression model if they modified the association between the exposure variables PO4 excretion and the outcomes epinephrine, norepinephrine, metanephrine or normetanephrine, i.e., if (i) changes in the β coefficient of predictors were ≥ 10%), (ii) they had an independent and significant effect on each outcome (*P* < 0.05), or (iii) they improved the explained variability of the model. Additionally, the backward elimination method was employed to refine and optimize the model. Variables were retained in the model if they met the predetermined significance level of *p* = 0.20. Finally, the sex-stratified multilple linear regression models were adjusted for the following covariates: (i) the growth period SDS means of FMI, 24-h urinary urea-nitrogen, urinary creatinine, urinary volume, urinary calcium and urinary potential renal acid load (uPRAL), and (ii) the blood parameters phosphate, creatinine, urea, albumin, uric acid, triglycerides, LDL, HDL glucose and insulin or HOMA-IR.

## Results

Table 1 provides a sex-stratified overview of the anthropometric and urinary excretion data at both youth and adult age as well as the blood parameters measured in adulthood.

**Table 1:** Characteristics of male (*n* = 73) and female (*n* = 86) participants during growth and adulthood^a^

Anthropometrics and urinary excretion data	Males		Females	
	Youth^b^	Adulthood	Youth^b^	Adulthood
Age, y	10.3 ± 0.9	18.3 (18.0, 22.4)	10.2 ± 0.8	21.0 (18.1, 22.7)
Weight, kg	40.6 ± 7.7	80.1 ± 15.1	37.2 ± 6.0	63.7 ± 11.2
Height, cm	145.1 ± 8.0	183.8 ± 6.0	141.6 ± 6.6	170.4 ± 5.6
BMI, kg/m²	18.0 ± 2.2	23.7 ± 4.4	17.4 ± 1.8	21.8 (19.6, 23.4)
BSA, m²	1.3 ± 0.1	2.0 ± 0.2	1.2 ± 0.1	1.7 ± 0.1
Urea-N, mmol/d	291.4 ± 59.0	457.2 (351.3, 544.3)	231.6 ± 42.2	305.9 ± 93.0
Urea-N (mmol/d/1.73 m²)	400.9 ± 55.6	391.1 (301.0, 465.5)	340.5 ± 48.3	303.8 ± 85.5
Volume, ml/d	926.3 (774.7, 1130.9)	1969.4 (1394.3, 2620.9)	965.1 ± 320.6	1840.0 (1260.0, 2428.9)
Volume (ml/d/1.73 m²)	1282.0 (1105.1, 1488.6)	1732.9 (1162.6, 2111.2)	1253.88 (1051.6, 1678.3)	1848.1 (1285.0, 2401.4)
Creatinine, mmol/d	7.3 ± 1.4	16.2 ± 3.6	6.0 ± 0.9	10.6 ± 2.3
Creatinine (mmol/d/1.73 m²)	9.2 ± 1.0	13.8 ± 2.6	8.2 ± 0.7	10.6 ± 1.9
Ca, mmol/d	1.5 (1.1, 2.1)	3.5 (2.6, 5.1)	1.8 ± 0.8	3.1 (2.2, 4.5)
Ca (mmol/d/1.73 m²)	2.3 ± 1.2	3.0 (2.2, 4.3)	2.5 ± 1.2	3.6 (2.3, 4.6)
uPRAL, mEq/d	14.1 ± 8.8	32.2 ± 36.7	5.6 ± 7.7	11.9 ± 24.4
uPRAL (mEq/d/1.73 m²)	17.9 ± 11.5	27.1 ± 29.8	7.5 ± 10.8	11.5 ± 23.3
Phosphorus (mg/d)^c^	1,088.3 ± 211.7	1654,5 ± 502.3	886.4 ± 147.7	1117.8 ± 320.07
Adulthood blood levels				
PO4 (mmol/L)		1.1 ± 0.2		1.1 ± 0.1
Creatinine (mg/dl)		0.9 (0.8, 1.1)		0.8 ± 0.1
Urea (mg/dl)		30.9 ± 8.3		23.4 ± 5.9
Albumin (g/L)		45.7 ± 2.0		44.0 (41.8, 46.3)
Glucose (mg/dl)		91.2 ± 8.5		89.0 (84.0, 94.0)
Insulin (mg/dl)		10.9 (7.8, 14.6)		11.1 (8.9, 13.7)
HOMA-IR		2.3 (1.7, 3.4)		2.7 ± 1.3
LDL (mg/dl)		87.0 (68.0, 113.0)		103.2 ± 30.3
HDL (mg/dl)		50.2 ± 9.6		64.0 ± 15.7
LDL/HDL ratio		1.9 ± 0.7		1.7 ± 0.7
Triglycerides (mg/dl)		97.3 ± 58.5		84.5 (66.0, 127.0)
Uric acid (mg/dl)		5.9 ± 1.0		4.4 ± 0.7

*BMI* body mass index; *BSA* body surface area; Urea-N, 24 h urinary urea nitrogen excretion; *Ca* calcium; *uPRAL* urinary potential renal acid load; *PO4* phosphate; *HOMA-IR* homeostasis model assessment-insulin resistance; *LDL* low-density lipoprotein cholesterol; *HDL* high-density lipoprotein cholesterol

^a^All values are means ± SDs if normally distributed or median (25th, 75th percentiles) if not normally distributed.

^b^Data denote means or medians of all individuals’ respective variable means.

^c^Estimated phosphorus intake calculated from urinary 24-h phosphate excretion under the assumption of an average intestinal absorption rate of 63%

cc

Among the 159 individuals, the average age for males (*n* = 73) was 10 years during growth and 18 years in adulthood. For females (*n* = 86), the average age was 10 years in youth and 21 years in adulthood.

**Fig. 2:**
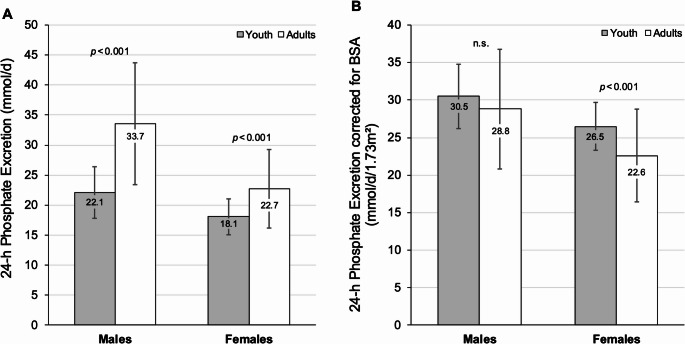
(**A**) 24-h urinary phosphate excretion in young and adult males and females and (**B**) 24-h urinary phosphate excretion corrected for body surface area (BSA) in youth and in adults, stratified by sex. Bars in (**A**, **B**) represent mean ± SD for youth and adults, sex stratified. Differences between youth and adulthood assessments were tested with the paired t-test for dependent normally distributed variables. All differences were statistically significant (*p* < 0.001), except for males’ 24-h urinary phosphate excretion corrected for BSA

Children’s and adolescents’ absolute 24-h urinary PO4-Ex markedly increased from youth to adulthood for both sexes (Fig. 2 A; *p* < 0.001). After correcting for an adult body surface area (BSA) of 1.73 m², PO4-Ex was 30.5 mmol/day/1.73 m² in males during youth and 28.8 mmol/day/1.73 m² in adulthood. In females, the corresponding values were 26.5 mmol/day/1.73 m² in youth significantly decreasing to 22.6 mmol/day/1.73 m² in adulthood (Fig. 2B).

Urinary potential renal acid load in males increased from 17.9 mEq/d/1.73m^2^ during youth to 27.1 mEq/d/1.73m^2^ in adulthood. In females, uPRAL was 7.5 mEq/d/1.73m^2^ in the early years, slightly rising to 11.5 mEq/d/1.73m^2^ in adulthood (Table 1).

**Fig. 3:**
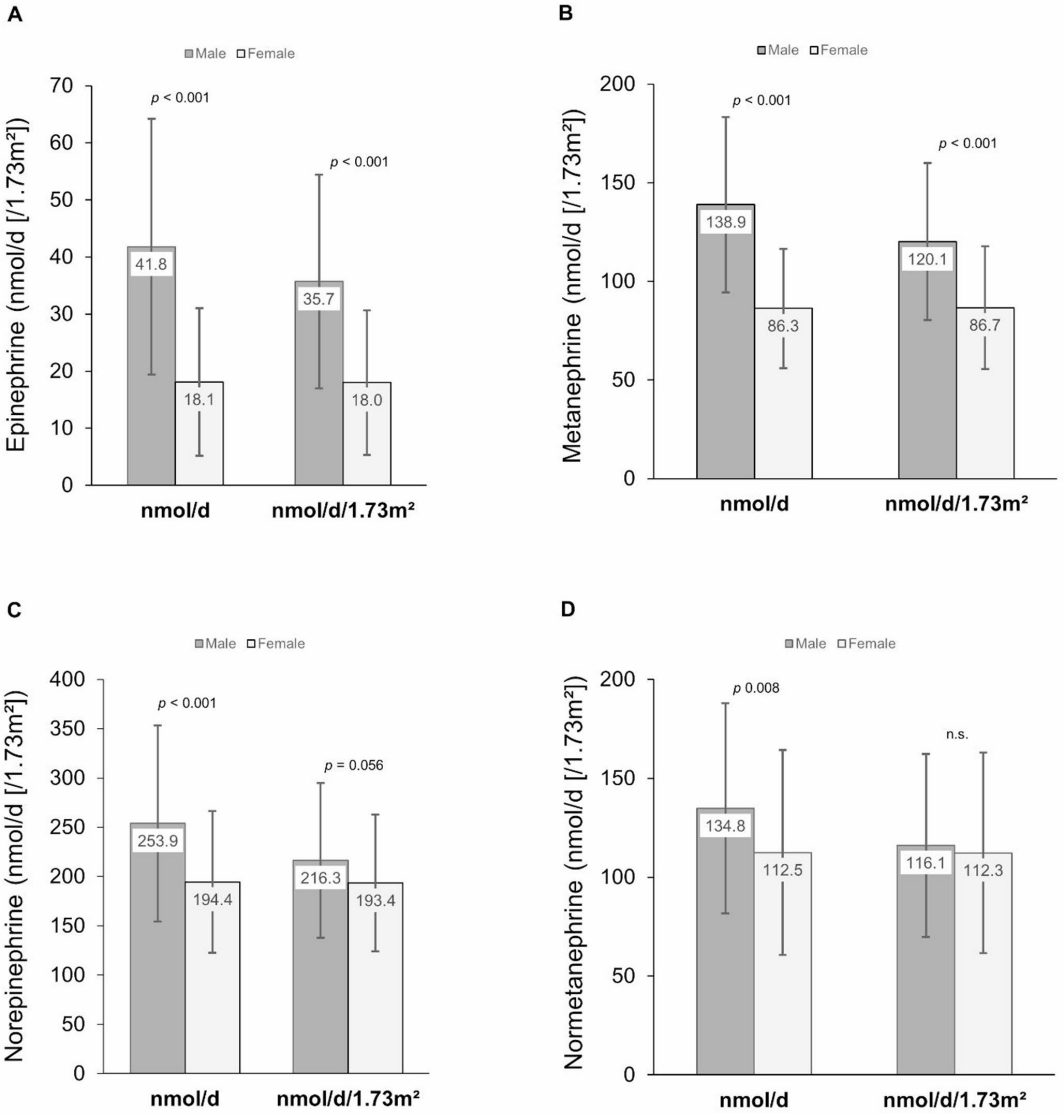
Urinary excretion of catecholamines in adulthood, stratified by sex. (**A**) 24-h excretion and body surface area (BSA)-corrected excretion of epinephrine (adrenaline) and its direct metabolite (**B**) metanephrine excretion in adulthood. (**C**) 24-h excretion and BSA-corrected excretion of norepinephrine (noradrenaline) and (**D**) its direct metabolite normetanephrine in adulthood. Bars in (**A**-**D**) represent mean ± SD for both male and female adults. Differences between males’ and females’ absolute 24-hour catecholamine excretion and BSA-corrected excretion were tested using the independent t-test for normally distributed variables. All differences were statistically significant (*p* < 0.001), except for norepinephrine excretion adjusted for BSA (*p* = 0.056) and normetanephrine excretion (n.s., non- significant)

Moreover, absolute 24-h excretion rates of the catecholamines EPI and NE and their metabolites differed significantly between males and females (Fig. 3). Mean EPI excretion in males was 41.8 nmol/d and 18.1 nmol/d in females (Fig. 3 A). NE excretion was 253.9 nmol/d and 194.4 nmol/d in males and females, respectively (Fig. 3 C). BSA-corrected excretion levels showed less clear sex differences with almost comparable excretions between males and female for normetanephrine (Fig. 3D).

The results of multilple linear regression analyses, examining the outcomes EPI and NE in relation to the exposure 24-h urinary PO4-Ex, are presented in Table 2. After specifically adjusting for nutrition-related urinary biomarkers (Model II), a close to significant association or a trend was observed for PO4-Ex in relation to EPI and NE in females. In the fully adjusted model, EPI demonstrated a significant positive relationship with PO4-Ex for females, but not for males (Table 2). Likewise, after full adjustment, NE associated significantly positively with PO4-Ex only in females.

**Table 2:** Prospective relationships of phosphorus intake – assessed by phosphate excretion during growth – with 24-h excretion rates of the adrenomedullary hormones epinephrine (adrenaline) and norepinephrine (noradrenaline)^a^

Outcome		Males Β (95% CI)	*R* ^2^	*P*	Females Β (95% CI)	*R* ^2^	*P*
Epinephrine (nmol/24h)	Model I^b^	8.8 (−0.7, 18.3)	0.06	0.069	3.2 (−2.6, 8.9)	0.02	0.275
	Model II^c^	12.5 (−6.4, 31.4)	0.39	0.191	7.4 (−0.8, 15.6)	0.29	0.077
	Model III^d^	5.2 (−4.6, 15.0)	0.39	0.296	7.9 (0.7, 15.1)	0.37	0.030
Norepinephrine (nmol/24h)	Model I^b^	28.5 (−14.3, 71.3)	0.02	0.188	18.8 (−12.5, 50.2)	0.02	0.236
	Model II^c^	6.9 (−82.9, 96.8)	0.32	0.878	39.4 (−9.9, 88.7)	0.37	0.115
	Model III^e^	27.5 (−12.1, 67.2)	0.50	0.169	45.2 (2.1, 88.3)	0.41	0.040

^a^Results obtained from stepwise multiple linear regression analyses stratified for males (m) and females (f)

^b^Model I adjusted for sex and means of SDSs of FMI during childhood and adolescence

^c^Model II adjusted for variables in model I plus all SDSs of nutrition-related 24-h urinary biomarkers gathered during (i) childhood and adolescence as well as (ii) adult urinary excretion that fulfilled the model inclusion criteria

^d^Model III adjusted for variables in model II plus adults’ blood-derived parameters. After removing all non-significant covariates with p-values exceeding 0.2, the final models III were adjusted for the following: SDSs of FMI (m, f), urea nitrogen (f), urine volume (m), PRAL (m), calcium (m, f), and adult urinary creatinine (m), urea nitrogen (f), calcium (f), and PRAL (f), as well as blood levels of albumin (m, f), creatinine (f), and phosphate (f).

^e^Model III adjusted for variables in model II plus adults’ blood-derived parameters. After removing all non-significant covariates with p-values exceeding 0.2, the final models III were adjusted for the following: SDSs of FMI (m, f), urea nitrogen (f), PRAL (f), calcium (f), adult urinary creatinine (m), urea nitrogen (m, f), volume (m, f) calcium (m, f), and PRAL (m, f), as well as blood levels of creatinine (m, f), uric acid (m), phosphate (m, f), LDL/HDL ratio (m), and triglycerides (m).

No significant associations were found between PO4-Ex and the excretions of the catecholamine metabolites metanephrine and normetanephrine (Table 3).

**Table 3:** Prospective relationships of phosphorus intake – assessed by phosphate excretion during growth – with 24-h excretion rates of the metabolites metanephrine and normetanephrine in adulthood^a^

Outcome		Males Β (95% CI)	*R* ^2^	*P*	Females Β (95% CI)	*R* ^2^	*P*
Metanephrine (nmol/24h)	Model I^b^	2.0 (−17.1, 21.2)	0.02	0.833	7.6 (−5.4, 20.7)	0.04	0.249
	Model II^c^	5.6 (−36.3, 47.5)	0.20	0.790	10.4 (−12.6, 33.5)	0.12	0.369
	Model III^d^	4.8 (−18.4, 28.1)	0.22	0.677	7.8 (−5.2, 20.8)	0.14	0.236
Normetanephrine (nmol/24h)	Model I^b^	5.8 (−17.3, 28.9)	0.007	0.617	13.8 (−8.7, 36.5)	0.02	0.225
	Model II^c^	−2.2 (−52.9, 48.5)	0.20	0.930	11.8 (−23.1, 46.8)	0.29	0.502
	Model III^e^	5.7 (−17.8, 29.1)	0.38	0.630	9.9 (−11.9, 31.8)	0.32	0.366

^a^Results obtained from stepwise multilple linear regression analyses stratified for males (m) and females (f)

^b^Model I adjusted for sex and means of SDSs of FMI during childhood and adolescence

^c^Model II adjusted for variables in model I plus all SDSs of nutrition-related 24-h urinary biomarkers gathered during (i) childhood and adolescence as well as (ii) adult urinary excretion that fulfilled the model inclusion criteria

^d^Model III adjusted for variables in model II plus adults’ blood-derived parameters. After removing all non-significant covariates with p-values exceeding 0.2, the final models III were adjusted for the following: SDSs of FMI (m, f), creatinine (m), calcium (f), and adult urinary creatinine (m), volume (m, f), as well as blood levels of phosphate (m), urea (m, f), HOMA-IR (m) and uric acid (f).

^e^Model III adjusted for variables in model II plus adults’ blood-derived parameters. After removing all non-significant covariates with p-values exceeding 0.2, the final models III were adjusted for the following: SDSs of FMI (m, f), calcium (m, f), adult urinary creatinine (m), urea nitrogen (f), volume (m, f), phosphate (f), and calcium (f) as well as blood levels of creatinine (m, f), uric acid (m), phosphate (m), and triglycerides (m, f).

## Discussion

This study aimed to determine whether a habitual high phosphorus intake in healthy children and adolescents may be prospectively related to higher sympathoadrenergic activity in adulthood. Girls’ and female adolescents’ phosphorus intake, assessed based on biomarkers via repeated measurements of 24-h urinary excretion rates of phosphate, proved to be significantly associated with the two major catecholamines secreted by the sympathoadrenal medullary system, namely, EPI and NE. Associations for boys were non-significant. The findings for females were in general accordance with the results of a 14-month high-phosphorus feeding study in mice reported by Latic et al. [10] and the results of a prospective out-patient study involving healthy human subjects ingesting a high phosphate intake for 11 weeks [9]. In both dietary intervention studies, which covered relatively long periods of high daily phosphate ingestion, significant phosphate intake-specific increases in sympathoadrenal activity were observed. However, in the human study, only urinary metanephrine and normetanephrine were measured—neither EPI nor NE had been assessed—and the significant results for both metanephrines were obtained without sex stratification [9]. One reason for the discrepant results between the latter study and ours regarding the relationships between dietary phosphate intake and metanephrine and normetanephrine levels might be that we measured the specific fractions of urinary free catecholamines, whereas Mohammad et al., in their 11-week study, determined total urinary metanephrines, comprising free and conjugated compounds after an acid hydrolysis.

The exclusive use of sodium phosphate salts over the 11 weeks for diet enrichment might have been another reason for the discrepancies between Mohammad et al.’s study (significant metanephrine-phosphate relationship) and our study (non-significant relationship for metanephrines).

Inorganic phosphate salts are not the usual natural sources of phosphorus intake and their increased ingestion for 11 weeks as well as their higher absorption rates [25] may not reflect a normal habitual adaption to a usual high phosphorus intake of which the surplus primarily does not consist of inorganic phosphate salts but mostly of organically bound phosphorus of processed and natural food. Thus, a more severe stimulus related to the higher oral sodium phosphate salt ingestion might have led to a higher transfer of the parent catecholamines to their non-functional O-methylated metabolites via the continuously metabolizing adrenalmedullary catechol-O-methyltransferase (Fig. 1), probably resulting in an increased release of metanephrine and normetanephrine.

Metabolism of EPI and NE to their respective O-methylated metanephrines within both pheochromocytoma tumor cells and normal adrenal medullary cells occurs virtually continuously and mostly independently of sympathoadrenal activity-related fluctuation in EPI and NE secretion. Similarly, sympathoadrenal activation in disease-free individuals with clear increases in EPI and NE secretion produces only small increases in free metanephrines compared with the parent amines [3]. The latter more or less continuous production of metanephrines is in line with our results following an obviously only moderate nutritional stressor, i.e., a long-term habitually high phosphorus intake resulting in merely moderate sympathoadrenal activation, with significant increases in EPI and NE but not yet clear increases in their O-methylated metabolites.

In the 14 month-long high-phosphate feeding study conducted in mice, all corresponding urinary catecholamines measured, i.e., metanephrine, normetanephrine, NE, and EPI, were elevated, although the latter did not reach full significance (*p* = 0.052) [10]. Overall this animal experimental finding based on an excessive sodium phosphate intake confirms that more pronounced stressor stimuli may induce increases of both the parent amines EPI and NE as well as their O-methylated metabolites. Unfortunately, the 11 week-long human dietary intervention study by Mohammad et al. [9] did not provide any measurement data of the diagnostically more conclusive catecholamines adrenaline and noradrenaline, i.e. of urinary free EPI and NE.

The mechanisms through which high nutritional availability of phosphate may trigger catecholamine secretion and due to which females may be more susceptible to habitually phosphorus-rich diets are not known. Whether higher phosphate availability in the circulation and thus a higher potential phosphate supply to the ATP-rich adrenomedullary tissue [4] may play some role in altering the secretory responsiveness of chromaffin medullary cells remains speculative [26]. What however should be mentioned with regard to potential nutritional-metabolic stimuli of EPI and NE release from the adrenal medulla is that all forms of acidosis are important triggers of increased catecholamine secretion [27–29]. In line with this fact, the dietary acid load PRAL, determined on the basis of biomarkers [18, 19] either during growth or in adult age, proved to be a significant covariate, adding to the total explained variance in the models for the outcomes EPI and NE (Table 2).

The question regarding why females rather than males showed enhanced catecholaminergic activity following habitual high dietary phosphorus intake cannot as yet be satisfactorily answered. Physiological differences between the sexes probably play a role, as evidence shows that catecholamine responses vary depending on the type of stressor: for example, women exhibit higher EPI and/or NEPI responses after alcohol ingestion [30, 31] or after specific ergometric exercises [32], whereas men respond with greater catecholamine increases after insulin-induced hypoglycemia [33] or mental/examination stress [22, 23]. Estrogens have been reported to attenuate catecholaminergic responses to various stress stimuli [34].

Catecholamines have metabolic hormonal effects at concentrations even slightly above low normal resting levels [7, 35], and with regard to sex differences, women in comparison to men exhibit increased cardiac-specific sympathetic activation, with possible consequences for their cardiovascular disease susceptibility [36].

Accordingly, the increased sympathoadrenal activity observed in females (Table 2) who have regularly ingested high amounts of phosphate and organically bound phosphorus in their diets may constitute one of the dietary and lifestyle factors involved in the development and/or maintenance of elevated blood pressure, respiratory dysfunction, mood and depressive disorders, and coronary heart disease [6, 37–39].

Interestingly, increased sympathetic nervous system activity along with an elevated NE level has very recently been identified in a number of sophisticated animal experiments as a critical driver in the pathogenesis of overnutrition-induced insulin resistance and metabolic disease, independent of cellular insulin signaling [40].

Due to the purely voluntary nature of participation in the DONALD study, the number of individuals who had regularly visited the study center during childhood and adolescence and then continued providing 24-hour urine samples and additional blood specimens in adulthood is rather limited. Accordingly, with a higher subsample size of male participants, an association between P-In and catecholamine excretion might have been observed also for males. Another limitation of the current study, apart from the relatively small sample size, is that the catecholamine outcomes were determined only once. It is also to be noted that the present findings, obtained in a cohort of children of European descent, are not necessarily applicable to other ethnicities. Furthermore, we were not able to differentiate between organically bound dietary phosphorus intake and phosphate salts added to food. This differentiation might have led to even clearer results, since especially the consumption of inorganic phosphate food additives, which have markedly higher bioavailability than natural phosphorus [25], can lead to more pronounced rises in blood PO4 concentrations [41].

Despite these limitations, the present study has specific strengths, one of which is its detailed sample and data collection, involving repeated 24-hour urine measurements over a long period. This allowed for adequate control of interacting covariates and inherent confounders. In particular, average daily acid loads during childhood and in the adult samples could be specifically adjusted using the integrative measurement variable urinary PRAL. Also of importance, we were able to adjust for adult blood phosphate concentrations, which might have additionally strongly influenced adult catecholamine secretion, independent of the long-term exposure of interest, i.e., the habitual dietary phosphorus intake during childhood and adolescence.

In conclusion, our study provides new, biomarker-based evidence that high phosphorus intake among healthy girls and female adolescents, despite being still in the usual phosphorus intake range, may be related to increased EPI and NE secretion in adulthood. This confirms animal and human experimental results suggesting that high P-In is linked to elevated catecholaminergic activity. In line with relatively low stressor responsiveness and largely continuous adrenal-medullary release of O-methylated metabolites under disease-free physiological conditions [3], no associations were seen between P-In and females’ metanephrine and normetanephrine secretion. However, the underlying mechanism(s) causing high P-In to become a potential EPI- and NE-related stressor particularly in females needs to be clarified in future research.

## Data Availability

The datasets generated and/or analyzed during the current study can be made available from theInstitute of Nutrition and Food Sciences, Nutritional Epidemiology, University of Bonn on reasonable request.
